# Strong “bottom‐up” influences on small mammal populations: State‐space model analyses from long‐term studies

**DOI:** 10.1002/ece3.2725

**Published:** 2017-02-12

**Authors:** John R. Flowerdew, Tatsuya Amano, William J. Sutherland

**Affiliations:** ^1^ Department of ZoologyUniversity of CambridgeCambridgeUK

**Keywords:** bottom‐up control, feeding experiment, long‐term population dynamics, masting, small rodents, state‐space model

## Abstract

“Bottom‐up” influences, that is, masting, plus population density and climate, commonly influence woodland rodent demography. However, “top‐down” influences (predation) also intervene. Here, we assess the impacts of masting, climate, and density on rodent populations placed in the context of what is known about “top‐down” influences. To explain between‐year variations in bank vole *Myodes glareolus* and wood mouse *Apodemus sylvaticus* population demography, we applied a state‐space model to 33 years of catch‐mark‐release live‐trapping, winter temperature, and precise mast‐collection data. Experimental mast additions aided interpretation. Rodent numbers in European ash *Fraxinus excelsior* woodland were estimated (May/June, November/December). December–March mean minimum daily temperature represented winter severity. Total marked adult mice/voles (and juveniles in May/June) provided density indices validated against a model‐generated population estimate; this allowed estimation of the structure of a time‐series model and the demographic impacts of the climatic/biological variables. During two winters of insignificant fruit‐fall, 6.79 g/m^2^ sterilized ash seed (as fruit) was distributed over an equivalent woodland similarly live‐trapped. September–March fruit‐fall strongly increased bank vole spring reproductive rate and winter and summer population growth rates; colder winters weakly reduced winter population growth. September–March fruit‐fall and warmer winters marginally increased wood mouse spring reproductive rate and September–December fruit‐fall weakly elevated summer population growth. Density dependence significantly reduced both species' population growth. Fruit‐fall impacts on demography still appeared after a year. Experimental ash fruit addition confirmed its positive influence on bank vole winter population growth with probable moderation by colder temperatures. The models show the strong impact of masting as a “bottom‐up” influence on rodent demography, emphasizing independent masting and weather influences; delayed effects of masting; and the importance of density dependence and its interaction with masting. We conclude that these rodents show strong “bottom‐up” and density‐dependent influences on demography moderated by winter temperature. “Top‐down” influences appear weak and need further investigation.

## 1 Introduction

The importance of food supply as a “bottom‐up” influence on vertebrate population growth, modified by “top‐down” processes (predators), as well as social interactions and stochastic disturbances (e.g., weather), has been integrated into a general theory explaining population abundance (Ostfeld & Keesing, 2000; Sinclair & Krebs, 2002). However, long‐term data sets, which are particularly valuable for investigating demographic‐environmental links (Frederiksen, Anker‐Nilssen, Beaugrand, & Wanless, 2013; Stenseth, 1995), are scarce (Boonstra & Krebs, 2012; Falls, Falls, & Fryxell, 2007; Flowerdew & Ellwood, 2001; Southern, 1970). We therefore have a generally poor understanding of the relative importance, and interactions, of food supply and weather on reproduction/growth rates and on interspecific differences in such processes in natural populations over long periods of time.

This long‐term (33 years) study of population growth and reproduction in bank voles *Myodes glareolus* and wood mice *Apodemus sylvaticus* concentrates on the importance of periodical heavy tree fruiting (“masting”), which is a common driver of woodland rodent dynamics (Flowerdew, 1993; Krebs, Cowcill, Boonstra, & Kenney, 2010; Mallorie & Flowerdew, 1994; Montgomery, Wilson, Hamilton, & McCartney, 1991; Watts, 1969). We also assess the impact of social interactions, weather and their interactions. We use precise measures of tree fruit‐fall (Flowerdew & Gardner, 1978), population density (a measure of social interaction), and winter temperature (c.f. Pucek, Jędrzejewski, Jędrzejewska, & Pucek, 1993; Flowerdew, 2007; Clotfelter et al., 2007; Falls et al., 2007). (Note, however, that the mechanistic understanding of climatic impacts on demography is limited by long causal chains and indirect effects; Krebs & Berteaux, 2006).

In essence, this study tests Krebs' (2013) “Hypothesis 4” which states that *The maximum standing crop* (of rodents) *at the peak of a rodent population fluctuation is directly related to the productivity of the species' food resources*. Krebs (2013) includes this as part of a suite of hypotheses (including “top‐down” influences) for understanding rodent population biogeography; he advocates testing this by experimentally increasing/reducing the food resources of a fluctuating population.

Earlier, Flowerdew and Gardner (1978) showed (by stomach contents analyses) that woodland fruits constituted much of the two rodents' diets; they also concluded that increased European ash, *Fraxinus excelsior*, fruit‐fall overwinter promoted survival and population growth of bank voles and, to some extent, wood mice. Here, we further test the influence of ash masting on rodent demography and how this may be modified by winter weather and population density (and their interactions). We assess numbers of rodents at their pre‐breeding trough and early winter peak density alongside the assessment of ash fruit production. We then use a state‐space modeling approach involving rodent numbers and environmental variables to explain among‐year variations in demographic parameters (c.f. Stenseth, VIljugrein, Jędrzejewski, Mysterud, & Pucek, 2002); this models the complex nonlinear dynamics while jointly estimating observation and process errors in the population time‐series data (Buckland, Newman, Thomas, & Koesters, 2004; Clark & Bjørnstad, 2004; Dennis, Ponciano, Lele, Taper, & Staples, 2006). In addition, we further test Hypothesis 4 with an experimental fruit‐addition study during 2 of 4 years live‐trapping nearby.

## 2 Methods

### 2.1 Study area

The study area in Lathkill Dale, Derbyshire ((SK 199 660), 241 m amsl (see Flowerdew & Gardner, 1978) had ash comprising 90% of the canopy in a stand 101–160 years old in 1967. The soil is stabilized scree with limestone bedrock often close to the surface, providing a highly favorable environment for both rodents.

### 2.2 Live‐trapping

Live‐trapping commenced in January 1971 and then usually in early June and early December until 2005. In all, 90 Longworth live‐traps, grouped in three within 1 m of each other, were placed at 30 (5 × 6) points spaced at 15 m, covering 0.45 ha. Trapping was carried out conforming to the English Nature guidelines concerning the Wildlife and Countryside Act (1981) (as amended). Traps were provisioned with hay, oats for rodents, and blowfly puparia for shrews. Traps positioned on Day 1 were pre‐baited for 48 hr; on Day 3, they were checked/re‐provisioned, cleaned, and set. Traps were visited in late afternoon Day 3, morning and afternoon Day 4, and morning Day 5. The afternoon and following morning captures of Days 3–4 and 4–5 were each amalgamated to provide two successive 24‐hr samples for population estimation. Exceptions are noted below. Until 1988, all rodents were individually marked on first capture by toe‐clipping or ear‐notching, and thereafter “session‐marked” by a single fur‐clip (unique for up to 2.5 years, J.R. Flowerdew, personal observation).

Six‐monthly sampling followed the peaks and troughs of annual population fluctuations (Flowerdew & Ellwood, 2001; Hansson, 1971; Southern, 1970). May/June trapping caught overwintered adults and usually young of the year. Young of both species from several litters usually appear during May–October/November (Harris & Yalden, 2008). The numbers of each species were counted for each 2‐day trapping period as the total adults newly marked or recaptured from a previous trapping period. Young were identified by mass (lower mode(s) than adults, usually separated by absent mass categories) and/or the presence of a greyish‐brown “juvenile” pelage. Totals of young were analyzed independently from adults (mean sample date 9th June, range: 5th–15th, except for 23 June 1973 and 29 May 2004). Any juvenile captures indicated that reproduction had started. In November/December, no evidence of winter breeding (pregnant/lactating females or recently weaned juveniles) was observed; this sample was treated as the overwintering adult population. Flowerdew and Gardner (1978) describe full trapping and handling procedures. Each 2‐day total of marked adult individuals was taken as an index of density (Slade & Blair, 2000) and validated against a population estimate generated by the state‐space model (assuming constant trappability over each trapping period) for use in the analysis of population growth rates and in estimating the structure of a time‐series model. In both species, trappability in adults is generally high with over 70% of the known individuals present being caught in the first 2 days of trapping (Gurnell, 1980).

### 2.3 Winter food supply measurement

Ash fruit‐fall was the major high‐energy food available. It was measured by 20 (5 × 4) dustbins (each having 0.166 m^2^ sampling space) secured at the center of each 15 m trapping grid square. Flowerdew and Gardner (1978) further describe sampling rationale and fruit assessment; parasitized fruits were removed to give “edible” totals over 33 years (see section 4). Each bin had a conical nylon netting insert secured by an aluminum band and several drainage holes (4 mm diameter). Fruits, leaves, etc., fell onto the netting and then through the 200‐mm diameter aperture at the apex (preventing fruit removal). From autumn 1971 to spring 2005, the bins were cleared (until no further fruit fell); usually from 1 September at approximately 6‐week intervals until 31st March or 30th April. Fruits were separated from leaves/debris; later, seeds were removed from the pericarp (wing) and dried at 80°C to constant mass. The dried (edible, non‐parasitized) seeds from each bin were counted and weighed together to the nearest 10th of a milligram. Mean early (September–November/December) and annual (September–March/April) fruit‐fall (as dry seed g/m^2^) were tabulated. Gardner's (1977) September–August collections of fruits/seeds (1966–1971) partly overlapped our study area and he found that little fell after April. Gardner's heaviest year (1969–1970, 998 seeds/m^2^) was equivalent to approximately 26.62 g/m^2^ dry mass (assuming 26.6766 mg mean mass per seed [see below]), somewhat higher than any from 1971 to 2005. Note that the adjusted (from zero) figure of 1.94 g/m^2^ for 1986–1987 was included after no fruits were observed in the canopy or bins; however, fruit and germinating seedlings were observed in April 1987, presumably from the heavy fruiting in 1985–1986 (embryo growth requires “stratification” at 5°C and germination usually occurs in the spring following fruit‐fall; Gardner, 1977). These remaining fruits/seedlings were presumably available as fruits in winter 1986–1987 and were estimated by searching until no more could be found in 20 × 0.5 m^2^ quadrats placed 1 m to the right of each bin. Total edible/unparasitized fruit and seedlings for each quadrat were multiplied by the mean mass of single dried seeds collected in 1985–1986 (26.6766 mg) and averaged, providing an estimated mean dry seed mass/m^2^. This is clearly a minimum estimate for 1986–1987. Fruits were also observed on the woodland floor (not quantified) during May/June–December following masting in 1996–1997, 1998–1999, and 2000–2001, and fruits/seedlings from 2003 to 2004 until May/June 2005.

### 2.4 Experimental addition of ash fruit

During October–March 1981–1982 and 1984–1985 (years of negligible natural fruit‐fall), 60 kg of heat‐sterilized ash fruit (treated at 80°C for 2 days) was distributed in the same monthly proportions observed in 1966–1967 (Gardner, 1977). Fruits were scattered fortnightly at the 5 × 4 (20) intermediate points 7.5 m from the nearest trapping points within a 6 × 5 point 0.45 ha grid, similar to, and circa 150 m west of, the main (control) area.

This experimental grid partially covered Area C (Gardner, 1977), being almost completely dominated by generally younger ash (66–100 years old in 1967; Merton, 1970); it had the same aspect, limited management and pattern of fruit‐fall as the long‐term grid, but a more mixed ground flora (Gardner, 1977). In 50 fruits sampled from the first sterilized fruit scattered, only two (4%) had aborted seeds, while the ratio of “dried mass edible/unparasitized seed” to “total fruit” was 0.5092 (G. Gardner, personal communication). Assuming the fruits were effectively scattered, they provided 6.79 g/m^2^ dry mass of edible/unparasitized seed, just below the two highest September–March/April fruit‐falls recorded from 1971 to 1981. Live‐trapping was synchronized with the control area during December 1981–June 1985 to compare rodent dynamics with additional ash fruit (1981–1982, 1984–1985) and without it (1982–1983, 1983–1984). Mice and voles were marked individually (see section 2.2).

### 2.5 Temperature measurements

Daily maximum and minimum temperature records from Buxton Meteorological Station (SK 4058E 3734N, 307 m amsl, circa 16 km NW, and 100 m higher, than Lathkill Dale) were obtained through the BADC service (see Acknowledgments). To measure winter severity, we used the mean December–March minimum daily temperature.

### 2.6 Statistical analysis for the observational data

Data used for state‐space modeling are the numbers of adult or juvenile individuals trapped (true abundances of each species at each life stage are unobservable—”hidden states,” in other words). State‐space models have two components: a process model and an observation model. Here, the process model comprised three parts, divided by two live‐trapping periods in June and December: (1) population dynamics from December to June, (2) reproduction completed by June, and (3) population dynamics from June to December. The overview of the model is shown in Figure S1.

The population dynamics from December of year *t* − 1 to June of year *t* were modeled using a discrete time, stochastic Gompertz model (Dennis & Taper, 1994):

(1) $$\begin{array}{cc} {\text{wgrowth}}_{t}\;=\; & \alpha_{\mathrm{wg}}+\beta_{\mathrm{wg},1}{\text{Ash}}_{\mathrm{mar},t-1}+\beta_{\mathrm{wg},2}{\text{Temp}}_{t-1}+\\ & \beta_{\mathrm{wg},3}\log\left({\text{Na}}_{\mathrm{dec},t-1}\right)+\beta_{\mathrm{wg},4}{\text{Ash}}_{\mathrm{mar},t-1}\log\left({\text{Na}}_{\mathrm{dec},t-1}\right)+\\ & \beta_{\mathrm{wg},5}{\text{Temp}}_{t-1}\log\left({\text{Na}}_{\mathrm{dec},t-1}\right)+\varepsilon_{\mathrm{wg},t} \end{array}$$

(2) $${\text{Na}}_{\mathrm{jun},t}∼\text{Poisson}\left({\text{Na}}_{\mathrm{dec},t-1}*\exp\left({\text{wgrowth}}_{t}\right)\right)$$

Here, Na_dec,*t* − 1_ and Na_jun,*t*_ are the numbers of adults in December of year *t* − 1 and June of year *t*, respectively. wgrowth_*t*_ represents the growth rate between the two periods, which is assumed to be affected by (1) ash fruit‐fall between September of year *t* − 1 and March of year *t* (Ash_mar,*t* − 1_), (2) mean daily minimum temperature between December of year *t* − 1 and March of year *t* (Temp_*t* − 1_), and (3) Na_dec,*t* − 1_. Interaction terms between Na_dec,*t* − 1_ and each of the other two factors were also included. Note that winter growth rate (December–June) is usually negative, reflecting winter mortality, but not necessarily restricted to being negative, to account for potential immigration. α_wg_ is the intercept and β_wg,1–5_ the coefficients. ε_wg,*t*_ is an error term following a normal distribution with zero mean and variance $\sigma_{\mathrm{wg}}^{2}$.

The reproduction completed by June of year *t* was modeled as follows:

(3) $$\begin{array}{cc} {\text{reprod}}_{t}\;=\; & \alpha_{r}+\beta_{r,1}{\text{Ash}}_{\mathrm{mar},t-1}+\beta_{r,2}{\text{Temp}}_{t-1}+\\ & \beta_{r,3}\log\left({\text{Na}}_{\mathrm{dec},t-1}\right)+\beta_{r,4}{\text{Ash}}_{\mathrm{mar},t-1}\log\left({\text{Na}}_{\mathrm{dec},t-1}\right)+\\ & \beta_{r,5}{\text{Temp}}_{t-1}\log\left({\text{Na}}_{\mathrm{dec},t-1}\right)+\varepsilon_{r,t}, \end{array}$$

(4) $${\text{Nj}}_{t}∼\text{Poisson}\left({\text{Na}}_{\mathrm{dec},t-1}*\exp\left({\text{reprod}}_{t}\right)\right)$$

where reprod_*t*_ represents the reproductive rate in year *t*, which is assumed to be affected by (1) Ash_mar,*t* − 1_, (2) Temp_*t* − 1_, and (3) Na_dec,*t* − 1_. Nj_*t*_ is the number of juveniles in June. Again interaction terms between Na_dec,*t* − 1_ and each of the other two factors were also included. α_*r*_ is the intercept and β_r,1–5_ the coefficients. ε_r,*t*_ is an error term following a normal distribution with zero mean and variance $\sigma_{r}^{2}$.

Finally, the population dynamics from June to December of year *t* were modeled similarly using a discrete time, stochastic Gompertz model:

(5) $$\begin{array}{cc} {\text{sgrowth}}_{t}\;=\; & \alpha_{\mathrm{sg}}+\beta_{\mathrm{sg},1}{\text{Ash}}_{\mathrm{mar},t-1}+\beta_{\mathrm{sg},2}{\text{Ash}}_{\mathrm{dec},t}+\\ & \beta_{\mathrm{sg},3}\log\left({\text{Na}}_{\mathrm{jun},t}+{\text{Nj}}_{t}\right)+\beta_{\mathrm{sg},4}{\text{Ash}}_{\mathrm{mar},t-1}\\ & \log\left({\text{Na}}_{\mathrm{jun},t}+{\text{Nj}}_{t}\right)+\beta_{\mathrm{sg},5}{\text{Ash}}_{\mathrm{dec},t}\log\left({\text{Na}}_{\mathrm{jun},t}+{\text{Nj}}_{t}\right)+\varepsilon_{\mathrm{sg},t}. \end{array}$$

(6) $${\text{Na}}_{\mathrm{dec},t}∼\text{Poisson}\left(\left({\text{Na}}_{\mathrm{jun},t}+{\text{Nj}}_{t}\right)*\exp\left({\text{sgrowth}}_{t}\right)\right)$$

Here, sgrowth_*t*_ represents the “summer” growth rate (June–December), which is assumed to be affected by (1) Ash_mar,*t* − 1_ (lagged effect of winter food supply in the previous year), (2) ash fruit‐fall between September and December of year *t* (Ash_dec,*t*_), and (3) the total number of individuals in June (Na_jun,*t*_ + Nj_*t*_). Interaction terms were similarly included. Note that the June–December growth rate is usually positive as reproduction occurs long after June. α_sg_ is the intercept and β_sg,1–5_ the coefficients. ε_sg,*t*_ is an error term following a normal distribution with zero mean and variance $\sigma_{\mathrm{sg}}^{2}$. The ash fruit‐fall and mean December–March daily minimum temperature (hereafter, “winter temperature”) were both standardized in all models to compare the relative importance of the two factors. log(Na_dec,*t* − 1_) in Equations 1 and 3 and log(Na_jun,*t*_ + Nj_*t*_) in Equation 5 are estimated, thus not able to be centered beforehand. Thus, models without interaction terms were first fitted to the data and the means of the estimated log(Na_dec_) and log(Na_jun_ + Nj) were estimated and then used to center these variables in the final interaction models.

The observation model also consists of three parts, each of which corresponds to the live‐trapping of adults in June, juveniles in June, and adults in December:

(7) $${\text{TrapNa}}_{\mathrm{jun},t,1}∼\text{Binomial}\left({\text{p.trapA}}_{\mathrm{jun}},{\text{Na}}_{\mathrm{jun},t}\right)$$

(8) $${\text{TrapNa}}_{\mathrm{jun},t,2}∼\text{Binomial}\left({\text{p.trapA}}_{\mathrm{jun}},\left({\text{Na}}_{\mathrm{jun},t}-{\text{TrapNa}}_{\mathrm{jun},t,1}\right)\right)$$

(9) $${\text{TrapNj}}_{t,1}∼\text{Binomial}\left(\text{p.trapJ},{\text{Nj}}_{t}\right)$$

(10) $${\text{TrapNj}}_{t,2}∼\text{Binomial}\left(\text{p.trapJ},\left({\text{Nj}}_{t}-{\text{TrapNj}}_{t,1}\right)\right)$$

(11) $${\text{TrapNa}}_{\mathrm{dec},t,1}∼\text{Binomial}\left({\text{p.trapA}}_{\mathrm{dec}},{\text{Na}}_{\mathrm{dec},t}\right)$$

(12) $${\text{TrapNa}}_{\mathrm{dec},t,2}∼\text{Binomial}\left({\text{p.trapA}}_{\mathrm{dec}},\left({\text{Na}}_{\mathrm{dec},t}-{\text{TrapNa}}_{\mathrm{dec},t,1}\right)\right)$$

Here, TrapNa_jun,*t*_ and TrapNa_dec,*t*_ are the number of adults trapped in June and December, respectively, and TrapNj_*t*_ is the number of juveniles trapped in June. The subscripts 1 and 2 indicate the captures of Days 3–4 and Days 4–5, respectively (see section 2.2). The captures of Days 4–5 excluded individuals already captured on Days 3–4. p.trapA_jun_, p.trapJ, and p.trapA_dec_ represent capture probabilities for adults in June, juveniles in June, and adults in December, respectively.

The model was fitted to the data with WinBUGS 1.4.3 (Lunn, Thomas, Best, & Spiegelhalter, 2000) and a package R2WinBUGS (Sturtz, Ligges, & Gelman, 2005) in R (R Core Team, 2012). Ash fruit‐fall and winter temperature were standardized before model fitting. Prior distributions of parameters were set as non‐informatively as possible. Gamma distributions with mean of 1 and variance of 1,000 were used as prior distributions for the inverses of $\sigma_{\mathrm{wg}}^{2}$, $\sigma_{r}^{2}$, and $\sigma_{\mathrm{sg}}^{2}$. Normal distributions with mean of 0 and variance of 1,000 were used as prior distributions for α_wg_, α_*r*_, α_sg_, β_wg,1–3_, β_*r*,1–3_, and β_sg,1–3_. Uniform distributions between 0 and 1 were used as prior distributions for p.trapA_jun_, p.trapJ, and p.trapA_dec_. A uniform distribution between 0 and 1,000 was used as a prior distribution for the number of adults in December of the first survey year (1971). Each Markov Chain Monte Carlo (MCMC) algorithm was run with three chains with different initial values for 10,000 iterations with the first 5,000 discarded as burn‐in. Model convergence was checked with R‐hat values (Gelman, Carlin, Stern, & Rubin, 2003) and trace plots of all the chains for sampling (Spiegelhalter, Thomas, Best, & Lunn, 2003).

### 2.7 Statistical analysis for the experimental data

To test the effects of the experimental addition of ash fruit (December 1981–June 1985) on mouse and vole population dynamics, demographic parameters in the experimental and control areas were estimated by fitting a simplified state‐space model. This model does not incorporate the effects of ash fruit‐fall, winter temperature, and density dependence on population dynamics, and consists only of Equations 2, 4, 6, and (7), (8), (9), (10), (11), (12). Normal distributions with mean of 0 and variance of 1,000 were used as prior distributions for wgrowth_*t*_, sgrowth_*t*_, and reprod_*t*_. Each MCMC algorithm was run with three chains with different initial values for 100,000 iterations, with the first 50,000 discarded as burn‐in and the remainder thinned to one in every ten iterations to save storage space. The longer iterations were due to relatively slow convergence compared to the main data model. Results provide winter growth rates (December–June), summer growth rates (June–December), and reproductive rates in June for the control and experimental grids.

## 3 Results

### 3.1 Environmental variables

**Figure 1:**
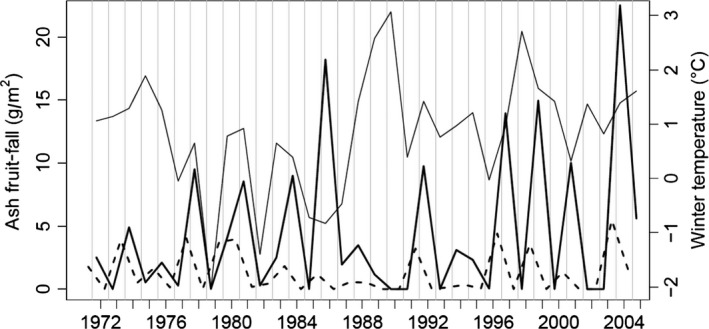
Ash fruit‐fall temporal dynamics (dry weight edible seed): September–March/April (thick solid line) measured yearly from 1971–1972 to 2004–2005; September–November/December (dashed line); winter temperature (see section 2) (thin solid line)

Winter temperature fluctuated greatly, but most winters were mild (range: −0.05 to 3.07°C, Figure 1). From 1971 to 1984, winter temperature was −0.05°C or above, except for −2.04°C in 1978–1979 and −1.4°C in 1981–1982. Then, during 1984–1987, all three winters fell below zero (−0.72, −0.83, and −0.47°C, respectively). Thereafter, winter temperature was warmer (range: −0.03 to 3.07°C). September–March ash fruit‐fall also fluctuated greatly, and early fruit‐fall (September–December) fluctuated similarly, but with lower values (Figure 1), suggesting that most fell during December–March. Particularly, heavy fruiting occurred in 1985–1986 and 2003–2004 (18.22 and 22.52 g/m^2^ edible/non‐parasitized seed, respectively); moderate production of 8.56–14.96 g/m^2^ occurred in seven other winters.

### 3.2 Bank voles

**Figure 2:**
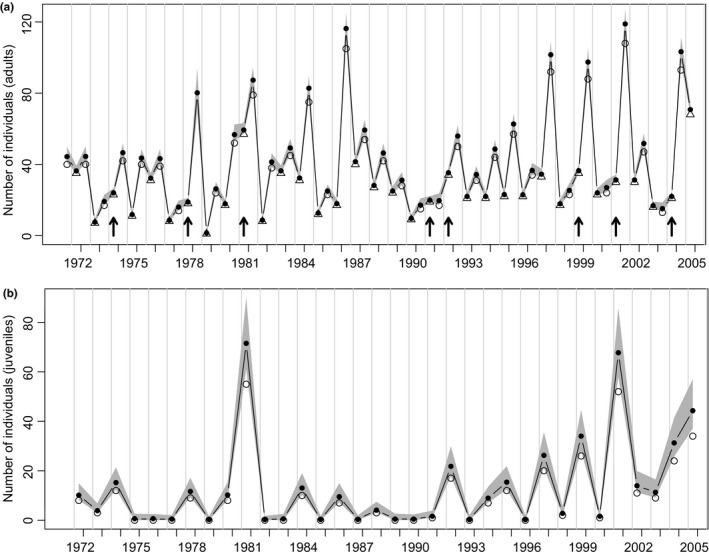
Bank voles: (a) Estimated numbers (filled circles); individual adults captured in May/June (open triangles); totals captured in November/December (open circles). Arrows indicate when (unusually) numbers increased from December to June. (b) Estimated (May/June) juvenile numbers (filled circles); individual juveniles captured (open circles). Note that the grey shading indicates the credible intervals of the estimated parameters. See section 2 for further details

The total number of adult bank voles trapped fluctuated greatly between years (range: 1–108); estimated (modeled) numbers were only slightly higher and both generally followed an annually cyclic pattern (Figure 2a).

**Figure 3:**
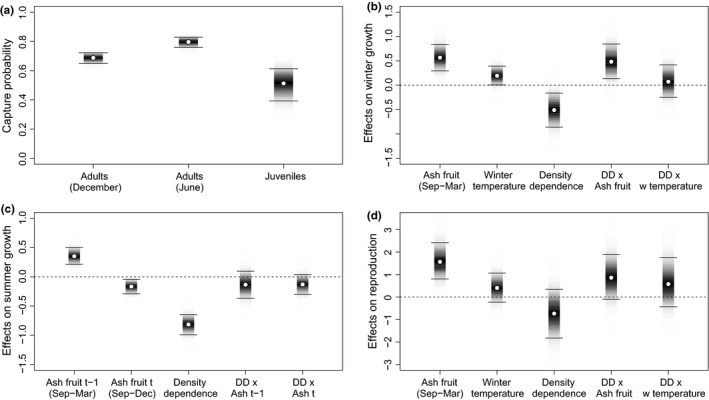
Bank voles: Estimated parameters. (a) Capture probabilities for adults and juveniles (May/June) and totals (November/December). (b) The effects on winter population growth rates (November/December–May/June) of ash fruit‐fall (September–March/April), winter temperature, density dependence and their interactions. (c) The effects on summer population growth rates (May/June–November/December) of previous ash fruit‐fall (September–March/April), current fruit‐fall (September–December), of density dependence and their interactions. (d) The effects on reproductive rate of ash fruit‐fall (September–March/April), winter temperature, density dependence and their interactions. Points represent median estimates, bars are shaded in proportion to the posterior probability density and horizontal lines mark 95% credible intervals

The estimated number of juvenile bank voles in May/June also fluctuated between years showing slightly higher values than the totals marked (Figure 2b) with median values below 72. Note the estimated capture probabilities were slightly greater in May/June than November/December and were higher in adults than juveniles (Figure 3a).

The estimated 95% credible intervals for all three parameters (ash fruit‐fall, winter temperature, and density dependence) affecting bank vole winter (December–May/June) growth rate (usually negative) did not overlap zero, indicating significant effects (Figure 3b). Overwinter (September–March/April) ash fruit‐fall and winter temperature had positive effects while density dependence had negative effects. Thus, the usual winter–summer decline in numbers may be reduced or reversed by elevated fruit‐fall or warmer winters; higher winter numbers decline more steeply than lower numbers due to density dependence. As the coefficient for ash fruit‐fall is slightly greater than that for winter temperature, the ash fruit abundance is judged to be the stronger influence. The estimated coefficient of the interaction term between ash fruit‐fall and density‐dependent effects on winter growth rate was significantly positive (Figure 3b); thus, the negative effect of density dependence is reduced by elevated fruit‐fall, while the positive effect of fruit‐fall becomes even stronger with higher winter numbers.

The estimated 95% credible intervals of all parameters affecting bank vole summer (May/June–December) growth rate (Figure 3c) did not overlap zero; thus, ash fruit‐fall in the previous winter positively affected summer growth, while concurrent ash fruit‐fall (September–December) and density dependence had negative effects. Thus, summer population growth rate is tempered by density dependence and moderately promoted (judged by model coefficient magnitude) by high fruit‐fall from the previous winter. September–December fruit‐fall, or more‐likely, a correlated variable (see section 4) had a barely significant negative influence on summer growth. Interaction terms were not significant. For the estimated spring reproductive rate, only September–March/April ash fruit‐fall had a 95% credible interval excluding zero, presumably promoting earlier starts to bank vole breeding and/or greater juvenile production by May/June, following high fruit‐fall (Figure 3d). Particularly high juvenile numbers occurred in early June 1981 and 2001 (Figure 2b), years following high, but not exceptional, fruiting. Interaction terms were again not significant.

### 3.3 Wood mice

**Figure 4:**
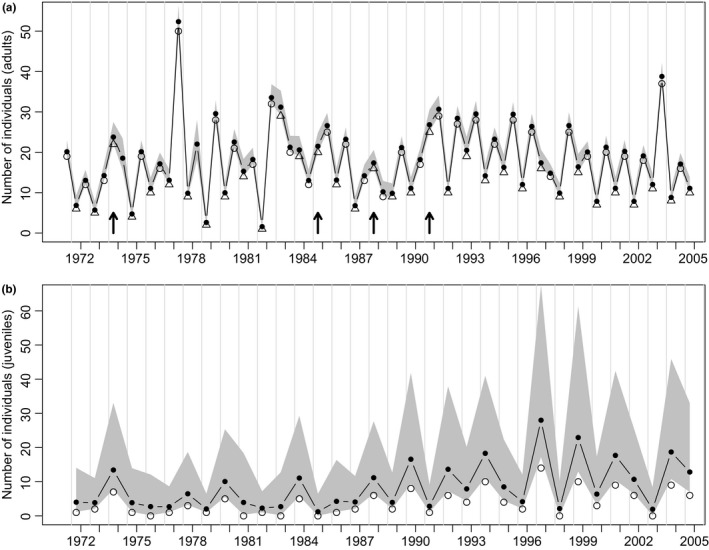
Wood mice: (a) Estimated numbers (filled circles); individual adults captured in May/June (open triangles); totals captured in November/December (open circles). Arrows, see caption for Figure 2. (b) Estimated (May/June) juvenile numbers (filled circles); individual juveniles captured (open circles). Note that the grey shading indicates the credible intervals of the estimated parameters. See section 2 for further details

**Figure 5:**
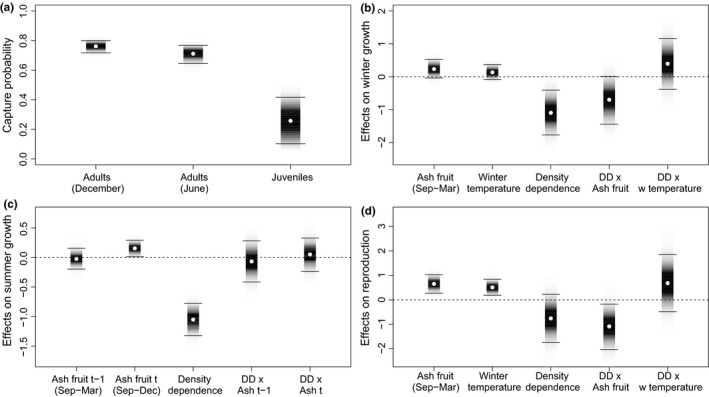
Wood mice: Estimated parameters. (a) Capture probabilities for adults and juveniles (May/June) and totals (November/December). (b) The effects on winter population growth rates (November/December–May/June) of ash fruit‐fall (September–March/April), winter temperature, density dependence and their interactions. (c) The effects on summer population growth rates (May/June–November/December) of previous ash fruit‐fall (September–March/April), of current fruit‐fall (September–December), and of density dependence and their interactions. (d) The effects on reproductive rate of ash fruit‐fall (September and March/April), winter temperature, density dependence and their interactions. Points represent median estimates, bars are shaded in proportion to the posterior probability density and horizontal lines mark 95% credible intervals

The trapped adult wood mouse numbers showed a lower fluctuating pattern than bank voles (range: 10–30), with model estimates slightly higher (Figure 4a). There was, however, a large difference between the numbers of juvenile wood mice estimated by the model and the numbers trapped (Figure 4b). This reflects the low juvenile capture probability (Figure 5a); adult capture probabilities were higher, being similar for both December and June.

The estimated 95% credible intervals for parameters affecting wood mouse winter (December–May/June) growth rate (usually negative) excluded zero only for density dependence (Figure 5b). Thus, higher winter populations showed greater declines than lower ones. There was, however, no significant impact on winter growth rate of overwinter total fruit‐fall or interaction terms. For summer growth rate of wood mice (Figure 5c), only con‐current ash fruit‐fall (September–December) and density dependence showed 95% credible intervals excluding zero, having barely significant positive and strongly significant negative effects, respectively; there was no significant effect of the previous winter's fruit‐fall or interaction terms. Thus, in wood mice, summer growth rate was strongly density dependent and only marginally improved by September–December fruit‐fall. Both September–March/April ash fruit‐fall and winter temperature had 95% credible intervals excluding zero in relation to reproductive rate (Figure 5d), both with marginally positive, similar, impacts. Thus, there was presumably earlier breeding and/or greater juvenile production by May/June following higher fruit‐fall and warmer winters. Although density dependence had a non‐significant influence on reproductive rate (95% credible interval overlapping zero), the estimated coefficient of the interaction term between ash fruit‐fall and density‐dependent effects on reproductive rate was significantly negative, suggesting that density dependence may have a greater negative effect under higher fruit‐fall, while the positive effect of fruit‐fall becomes weaker with higher population numbers.

### 3.4 Effect of experimental ash fruit addition

**Figure 6:**
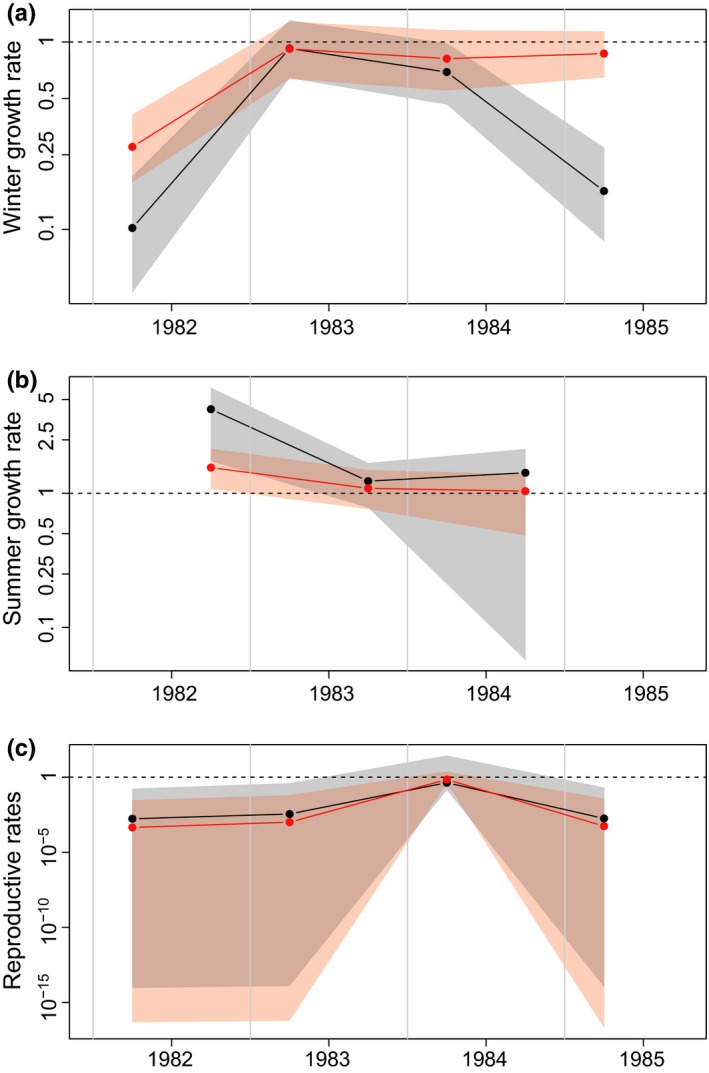
(All log scales). Bank voles: (a) Estimated winter population growth rates shown as the exponential of wgrowth_*t*_ (November/December–May/June), (b) summer growth rates shown as the exponential of sgrowth_*t*_ (May/June–November/December), and (c) reproductive rates in control and experimental areas shown as the exponential of reprod_*t*._ Black dots with grey areas and red dots with red areas represent the median estimates with the 95% credible intervals for control and experimental areas, respectively

**Figure 7:**
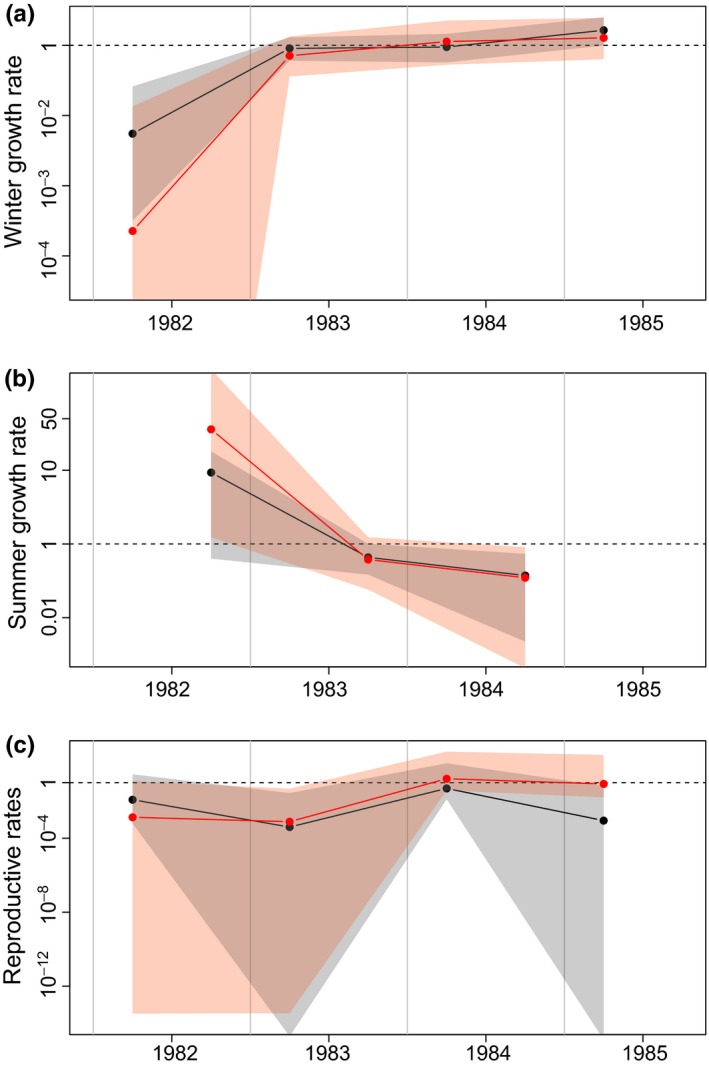
(All log scales). Wood mice: (a) Estimated winter population growth rates shown as the exponential of wgrowth_*t*_ (November/December–May/June), (b) summer growth rates shown as the exponential of sgrowth_*t*_ (May/June–November/December), and (c) reproductive rates shown as the exponential of reprod_*t*_ in control and experimental areas. Black dots with grey areas and red dots with red areas represent the median estimates with the 95% credible intervals for control and experimental areas, respectively

In bank voles, the estimated winter growth rate (usually negative) was greater on the experimental grid than control grid in both 1981–1982 and 1984–1985 (Figure 6a), the years of fruit addition (95% credible intervals barely overlapping or completely non‐overlapping). Furthermore, winter growth rate was similar on both areas in the intervening years without fruit addition (further indicating that the two areas were indeed very similar, see section 2). Thus, the similarity of the results for bank vole winter growth rate with additional food in 1984–1985 (Figure 6a) to those from the previous 2 years with natural food available (2.5 and 9.0 g/m^2^ respectively; Figure 1) further emphasizes the strong influence of the additional food in this winter when no natural ash fruit‐fall occurred. The closer values of winter growth rate in 1981–1982 (with and without additional ash fruit) compared with 1984–1985 may reflect the impact of the very severe winter temperatures on both areas in 1981–1982 (second lowest observed between 1972 and 2004) in comparison with the milder 1984–1985 (Figure 1). Thus, the ash‐fruit addition effects on bank vole winter growth support the conclusions from the long‐term data with a further suggestion that masting has a smaller influence on winter growth during cold winters. Bank vole summer growth rates and spring reproductive rates were similar on the two areas in all 4 years (Figure 6b,c). Wood mouse winter growth rates were similar on the two areas, as were their summer growth rates and spring reproductive rates throughout the experiment (Figure 7a–c).

## 4 Discussion

Our state‐space models indicate that winter masting, winter temperature, and population density and some interactive effects, each played significant, but contrasting, roles in driving the demography of these woodland rodents. In wood mice, there were weak influences of masting on spring reproductive rate and of September–December fruit‐fall (negatively) on summer growth rate. Winter severity reduced bank vole winter growth rate and wood mouse spring reproductive rate. Density dependence significantly affected both species' winter and summer population growth rates. Experimental data confirmed the masting influence on bank vole winter population growth rate and provided further insight into the influence of winter temperature.

We therefore provide substantial support for Krebs' (2013) “Hypothesis 4” (see section 1) concerning “bottom‐up” influences on populations, linked with important roles for density dependence and winter temperature. In measuring winter food supply, our assessment of “available food” should be considered as a maximum because: a) there may be alternative ash fruit predators (e.g., bullfinches, *Pyrrhula pyrrhula*; Greig‐Smith & Wilson, 1985), although Gardner (1977) could find no evidence for non‐rodent predation, and b) there is a possibility (particularly for bank voles which increase in trap captures with ground cover; Southern & Lowe, 1968) that an individuals' distance from cover increases their perceived predation risk (Cresswell, Lind, & Quinn, 2010) and so some fruit may be ignored even when theoretically available.

Food supply and winter temperature had independent effects on bank vole dynamics. This contrasts with the state‐space model results for bank voles of Stenseth et al. (2002) where oak masting influenced winter density but NAO (North Atlantic Oscillation), their proxy for climatic conditions, only weakly affected winter density. The latter relationship was further explained by food supply as NAO influence disappeared from their model when food supply was incorporated.

In both species, the adult probability of capture was higher in both November/December and May/June than for juveniles in May/June, especially for wood mice (Figures 3a and 5a). This may be the result of a variety of methodological, climatic, or biological influences (Gurnell & Flowerdew, 2006); we suggest that the reduced trapping of juveniles may be because they are naïve to traps so lack experience of any attraction of bait (Cole & Batzli, 1978) and may also be more “neophobic.”

The September–March ash fruit‐fall strongly affected winter growth rate, spring reproductive rate, and the subsequent summer growth rate (Figure 3b–d). This agrees with many UK and European bank vole studies which show a delay (to the following year) in the population response to masting (Crespin et al., 2002; Jensen, 1982; Kühn, Reil, Imholt, Mattes, & Jacob, 2011; Mallorie & Flowerdew, 1994; Pucek et al., 1993; Smyth, 1966; Watts, 1969). Here, we provide further evidence from precise fruit‐fall data that the bank vole population reaction to masting culminates in strong winter and summer growth and strong reproduction, leading to a population peak in the winter following the mast peak. Winter growth is further modified by winter temperature and density dependence also strongly affects growth, especially in summer. Interaction terms in the bank vole model indicate that the negative effect of density dependence on winter growth is reduced by elevated fruit‐fall, while the positive effect of fruit‐fall becomes elevated with higher winter numbers.

Surprisingly, our model suggests that September–December fruit‐fall had a weak negative influence on concurrent bank vole summer growth (Figure 3c). This is counter‐intuitive, being previously non‐significant in similar analyses (Flowerdew, 2007; Mallorie & Flowerdew, 1994). We suggest this may be explained by a correlate of low fruit‐fall; perhaps fruit remaining from heavy fruiting in the previous year (Figure 3c). Surplus fruit commonly remained on the woodland floor following winters with high fruit‐fall (8.5–22.52 g/m^2^ dry seed mass [Figure 1]) (see section 2); these high fruit‐fall winters were usually followed by low fruit‐fall the following year. Thus, the negative effect of September–December ash fruit‐fall (Ashdec,t) may actually represent a part of the positive effect of fruit‐fall from the previous year (Ashmar,*t* − 1) that was not reflected in our measure of Ashdec,t.

In bank voles, there was a strong influence of fruit‐fall but not of winter temperature on spring reproductive rate (Figure 3d), while these variables affected wood mouse reproductive rate equally (Figure 5d). However, there was no evidence of winter breeding in either species in November/December, even when the following spring reproductive rate was high and May/June juveniles (numbers and mass) indicated that breeding had started earlier than usual. It is possible that the prolonged masting of ash may not be as effective in stimulating small rodent reproduction throughout the winter, as occurs periodically in the short, but intense, masting of oak *Quercus* spp. and beech *Fagus* spp. (Jensen, 1982; Pucek et al., 1993; Shimada & Saitoh, 2006; Smyth, 1966; Wolff, 1996). (N.B. oak woodland may produce up to 64.54 g/m^2^ dry mass of acorns over a short period in 1 year; Tanton, 1965).

In wood mice, masting and temperature impacts on spring reproductive rate compare well with Polish observations where autumn seed crop and, to a lesser extent March mean daily temperature, explained 78% of the variation in mean juvenile abundance of yellow‐necked mice *Apodemus flavicollis* in April (Pucek et al., 1993). However, the strongly significant influence of ash fruit and the (just) non‐significant influence of winter temperature on bank vole reproductive rate in the current study contrasts with Pucek et al. (1993) who concluded that early breeding was related only to warmer winters. Other factors may also influence the start and intensity of bank vole breeding in spring: Eccard and Ylönen (2001) observed that a high density (of females) in spring delayed breeding despite high levels of experimental food being available (i.e., density‐dependent effects on breeding negate any effect of masting). In the current study, the density‐dependent influence on wood mouse reproductive rate was non‐significant. However, our wood mouse interaction terms between ash fruit‐fall and density‐dependent effects on reproductive rate suggested that density dependence may have a greater (and significant) negative effect under higher fruit‐fall, while the positive effect of fruit‐fall becomes weaker with higher individual population numbers, contrasting with the interactions seen with bank voles. Further work on interactions between temperature, food supply, and population density in their effects on reproduction is needed to help explain these contrasts, especially as in the present study the variation in the timing of the period of sampling (see section 2) may have affected the numbers of juveniles captured, allowing only an approximate annual assessment of the intensity of breeding and early production of young.

In the current study, winter temperature had a moderate, but less significant, impact than fruit‐fall on bank vole winter growth, whereas neither variable had a significant effect on wood mouse winter growth (Figures 3b and 4b). In contrast, pedunculate oak *Quercus robur* and ash fruit‐fall had a weaker influence than winter temperature on adult bank vole numbers in May in Cambridgeshire (Flowerdew, 2007). Our model results for wood mice contrast even more with the Cambridgeshire study, which showed strong effects of fruiting and only slightly less strong effects of winter temperature on adult numbers in May (Flowerdew, 2007).

In addition, early (September–December) fruit‐fall had a marginally significant positive effect on wood mouse summer growth (Figure 5c). This agrees with many UK and European wood mouse studies that show an immediate effect of masting on winter numbers (and spring reproduction), but contradicts many others where there is also a significant effect of masting on overwinter population growth (Flowerdew, 1976; Jensen, 1982; Mallorie & Flowerdew, 1994; Margaletic, Glavaš, & Bäumler, 2002; Watts, 1969). However, in our model, the effect of ash fruit on winter growth was only just insignificant (Figure 5b). This lack of a strong impact of masting on winter growth rate is curious and it may be that male wood mice react differently to females with respect to increased ash fruit availability. In the first 7 years of our study, there was a positive effect of masting on overwinter survival only for wood mouse females (Flowerdew & Gardner, 1978), which has not been further tested; such sex‐specific relationships are not unusual in experimental food‐addition studies (Fordham, 1971; Galindo‐Leal & Krebs, 1998).

Density dependence played an important role in influencing the dynamics of adult populations of both rodents, having strong effects on both winter and summer growth rates (Figures 3b,c and 5b,c), but not on reproduction (Figures 3d and 5d). Such widespread effects of density dependence, regulating (sensu Sinclair & Pech, 1996) and stabilizing numbers are common in these rodent genera, although they vary in strength between seasons, species, and location, with territoriality, competition for food, reduced pregnancy rates, and delayed maturation being cited as probable causes (Mallorie & Flowerdew, 1994; Montgomery, 1989a,b; Saitoh, Bjørnstad, & Stenseth, 1999; Stenseth et al., 2002; Watts, 1969). Experimental manipulations of wood mice (Montgomery, Wilson, & Elwood, 1997) showed that spatial density‐dependent inhibition of female breeding regulates summer–winter population increase. However, this mechanism may be overridden by superabundance of food in some years.

The relatively moderate density of ash fruit in the current study did have a positive effect on spring reproductive rates; in Figures 3d and 5d, we show that young of the year of both species represented a higher proportion of the winter adult population after higher fruit‐fall, which was probably caused by bringing forward the start of reproduction as well as enabling greater early pregnancy success. This allowed numbers of bank voles, and to some extent wood mice, to increase in the year following masting. It is perhaps not surprising that continuous winter breeding was not evident for the time‐period/seed mass reasons in ash masting stated above. Furthermore, the precise nutritional (and therefore calorific) value of ash fruit is in doubt because of (a) the possible influence of plant secondary chemicals (e.g. phenols), which interfere in protein digestion (Smallwood & Peters, 1986), and (b) the variation in such chemicals between fruits or trees (Greig‐Smith & Wilson, 1985). In addition, differences in sensitivity to tannins have been suggested as one possible cause of varied responses to masting in small rodents (Shimada & Saitoh, 2006).

Our experimental addition of ash fruit (6.79 g/m^2^ over October–March) mimicked, especially in the warm winter of 1984–1985 (Figure 6), the influence of masting on bank vole winter growth when fruits were available at that level and above. This suggests that masting effects on bank vole winter growth are modified by winter temperature and that relatively low fruit‐fall (in comparison with other woodland fruit‐fall measurements [see above]) will still have a significant impact on winter growth. The experiment showed no significant differences in summer growth or reproductive rate of bank voles as well as great variation in their demographic coefficients, possibly reflecting small sample sizes and low temporal/spatial replication. However, the similarity of demographic parameters within both species during the non‐ash fruit addition years (1982–1983 and 1983–1984) suggests that on both areas fruit‐fall, environmental conditions and habitat were indeed similar in these years, emphasizing the significance of the differences found between the areas for bank voles in the experimental years.

The possibility of some “top‐down” influence by predators (see section 1) has not been ruled out. However, logistical problems prevented the study of both Tawny owls (*Strix aluco*) and weasels (*Mustela nivalis*) which were present on the study area(s) (J.R. Flowerdew, personal observation). Where such predators have been studied (in comparable temperate deciduous woodlands), weasels appear to have no observable effect on the survival or density of either prey species (King, 1980), and data on their combined predation support the idea that inversely density‐dependent predation by owls and possibly by weasels could help to produce the kind of population fluctuations observed in small mammal studies (Southern & Lowe, 1982). Furthermore, in Bialovieza Forest, Poland, (Jędrzejewski, Jędrzejewska, & Szymura, 1995), even with the presence of a range of generalist predators as well as high densities of specialist weasels accounting for most of the winter mortality of bank voles and yellow‐necked mice, *Apodemus flavicollis*, their combined predation did not prevent rodent numbers from fluctuating wildly between years in response to variations in masting.

This study shows “bottom‐up” impacts on the bank vole population of winter masting on summer growth and on spring reproductive rate, as well as density dependence in both winter and summer growth and lesser impacts of winter temperature on winter growth. The impacts on the wood mouse population were limited to strong density dependence on winter and summer growth with weak influences of early winter food supply on summer growth; spring reproductive rates were only marginally significantly affected by both food supply and winter temperature. Corroboration of these relationships from the overwinter addition of ash fruit is only present for bank vole winter growth, but overall, “bottom‐up” influences on these small rodent dynamics are evidently very strong. Although this generally supports Krebs' (2013) Hypothesis 4, we still acknowledge that “top‐down” influences may play a large part in small mammal population dynamics elsewhere (Matson & Hunter, 1992; Korpimäki, Brown, Jacob, & Pech, 2004; Meserve et al., 2003; King & Powell, 2007). Here, their influence appears weak, much as Jędrzejewski et al. (1995) reported for Bialowieza in Poland, and these contrasts require further investigation.

## Data Accessibility

Data available from The Environmental Information Data Centre http://doi.org/10.5285/80ee4e00-7301-4c40-9dba-12dd0d21b7c7 (Flowerdew, Amano, & Sutherland, 2015).

## Conflict of Interests

None declared.
