# Supplementary material for: Strong “bottom‐up” influences on small mammal populations: State‐space model analyses from long‐term studies
Source: Ecol Evol. 2017 Feb 12;7(6):1699–711. doi: 10.1002/ece3.2725 (PMC5355190; doi:10.1002/ece3.2725)
Supplement: Supplementary file 1 [file ECE3-7-1699-s001.pdf]

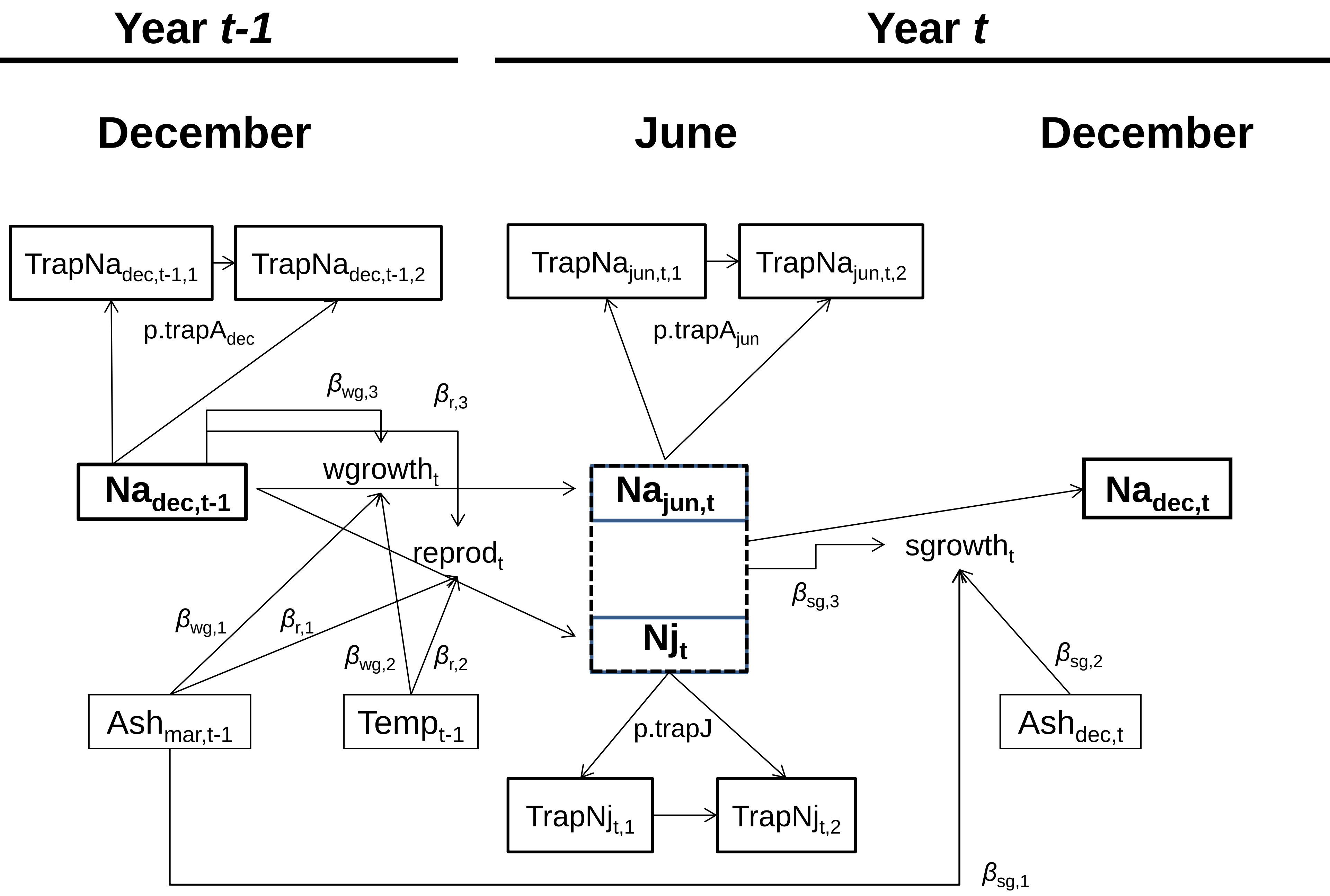

Year t-1
Year t
December
June
December
TrapNa$_{dec,t-1,1}$
TrapNa$_{dec,t-1,2}$
TrapNa$_{jun,t,1}$
TrapNa$_{jun,t,2}$
p.trapA$_{dec}$
p.trapA$_{jun}$
$\beta_{wg,3}$
$\beta_{r,3}$
Na$_{dec,t-1}$
wgrowth$_t$
Na$_{jun,t}$
Na$_{dec,t}$
reprod$_t$
sgrowth$_t$
$\beta_{sg,3}$
$\beta_{wg,1}$
$\beta_{r,1}$
Nj$_t$
$\beta_{wg,2}$
$\beta_{r,2}$
$\beta_{sg,2}$
Ash$_{mar,t-1}$
Temp$_{t-1}$
p.trapJ
Ash$_{dec,t}$
TrapNj$_{t,1}$
TrapNj$_{t,2}$
$\beta_{sg,1}$

Definition of parameters

$Na_{dec,t-1}$: no. adults in December of Year t-1
$Na_{jun,t}$: no. adults in June of Year t
Nj_t: no. juveniles in June of Year t

$TrapNa_{dec,t,1}$, $TrapNa_{dec,t,2}$: no. adults trapped on the 1st and 2nd days in December of Year t
$TrapNa_{jun,t,1}$, $TrapNa_{jun,t,2}$: no. adults trapped on the 1st and 2nd days in June of Year t
$TrapNj_{t,1}$, $TrapNj_{t,2}$: no. juveniles trapped on the 1st and 2nd days in June of Year t

$p.trapA_{dec}$: trapping efficiency for adults in December
$p.trapA_{jun}$: trapping efficiency for adults in June
p.trapJ: trapping efficiency for juveniles in June

$wgrowth_t$: growth rates between Dec and Jun (including survival and immigration)
$sgrowth_t$: growth rates between Jun and Dec (including survival, reproduction and immigration)
$reprod_t$: reproductive rate between Dec and Jun

$Ash_{mar,t-1}$: Ash fruit density between Sep of year t-1 and Mar of year t
$Ash_{dec,t}$: Ash fruit density between Sep and Dec of year t
$Temp_{t-1}$: mean daily minimum temperature between Dec of year t-1 and Mar of year t
